# Chloral Hydrate

**Published:** 1881-01

**Authors:** H. H. Kane

**Affiliations:** New York


### ARTICLE VI.

CHLORAL HYDRATE. (Article II.) By H. H. KANE, M.D., New York.

*Mode of Administration.*—The first duty of a conscientious physician who finds it necessary to use a drug of the strength of chloral, is to assure himself that the drug he has, or the article he orders from the apothecary, is pure. As has already been said, some authorities believe that the evil effects occasionally following the use of chloral are due to impurities in the drug, and not to peculiarities incident to the individual. This is especially insisted on by Liebreich and Bouchut,* and the latter gives the following simple test as sufficiently reliable for determining roughly the purity or impurity of the drug: "By taking it (chloral) in a crystallized, needle-shaped or snow-like mass, it will, in all probability, be found acceptable, but if you wish to insure its purity, you must test it with a concentrated solution of potash. If the chloral hydrate is pure, it will color the potash solution possibly a very light yellow, and will emit an unmistakable odor of chloroform. If absolutely pure, the solution should remain uncolored. If a brown color shows itself, and there is given forth the odor of chloroform, mixed with chloro-acetic

* Bouchut, C'te Rendu, Acad. des Scien. 1869, p. 966.

vapors, it is impure. I will say the same if it emits irritating gases of an acrid and disagreeable smell."

The fact that chloral hydrate is to be seen at different times and in different stores in several different conditions, has created not a little confusion in the professional mind. As manufactured by Schering, it comes to us perfectly pure, in white, opaque, broken plates of from about $\frac{1}{6}$ to $\frac{1}{4}$ in. in thickness. If this stands for a short time in a cool place, and is imperfectly corked, it will break down into a snowy mass. If freer access is given to the air, and especially if exposed to light and heat, the cake chloral becomes moist, of a yellow color, breaks down and very fine needle-shaped crystals form on the side of the bottle furthest from the heat. I have repeatedly tried to produce these shapes, and have always succeeded. Chloral in any of these forms may be perfectly pure, for I have tested it in each form and found it so. The needle-shaped crystal is that form most likely to be acrid and irritating, and if the process of its formation has been rapid, it will often give a distinct acid reaction and emit an acrid odor. There is, of course, a difference in the activity and the irritating qualities of these several crystalline forms, but by observing the precautions about to be given, all irritation may be avoided, and the difference in activity is too slight to need righting.

Chloral hydrate may be given by the mouth, rectum or hypodermically, or by the intra-venous method. I give them in the order of desirability. By the rectum we get about the same effect as by the mouth, both as regards rapidity of action and intensity and duration of effect. By the subcutaneous plan there is always danger of the production of deep-seated and superficial inflammation and the formation of abscess. The intravenous method is unjustifiable, save in the very rarest instances, and is fraught with great danger. In the majority of cases, then, the drug should be given either by the mouth or rectum.

*By the Mouth.*—Chloral hydrate should always be given in solution, as the drug in crystal is extremely irritating. Dr. Squibb prefers giving it in plain water, he believing that the syrup that is usually given to disguise the taste of the drug favors its breaking up. This plan is favored by not a few of my correspondents. A preparation known as Liebrich's syrup of chloral is advertised and extensively used in England. In giving chloral I have always found it most convenient to order a simple aqueous solution, say:

℞ Chloral hydrat.................................. ʒiv

Aq.............................................. ℥iij of which one drachm contains ten grains, and then ordering separately a bottle of syr. tolut or prunus virginian, and let the patient add a drachm or two of the syrup to each dose of the chloral solution at the time of taking.

Many physicians use the bromide of potassium with chloral in every instance, they believing that the former intensifies and prolongs the effect of the latter.

Bartholow* found good effects in nervous disease from a combination of chloral, morphine and atropine; better than when either drug was used singly.

Bowers† produced profound sleep, lasting from twelve to eighteen hours with the following:

| | | | |
|---|---|---|---|
| ℞ | Chloral hydrat. ................. | gr. 30 | a dose. |
| | Potass. bromid. ................ | gr. 15 | |
| | Tr. opii ........................ | gtt. 20 | |

Nearly the same is advocated by Dr. J. M. Lewis, of Kosciusko, Miss., who advocates the following, which he uses to produce sleep and quiet pain in every case where there are no contra-indications:

℞ Morphiæ sulph........................... gr. ¼

Chloral hydrat........................... gr. 15

Repeat as often as necessary to relieve pain or produce sleep. occasionally combines it with potass. bromid.

With reference to the combined use of chloral hydrate and morphia, Bartholow‡ says:

"These agents act much more favorably when administered simultaneously. Chloral causes sleep, morphia relieves pain,

* *Cincinnati Clinic*, Oct. 28, 1874.

† *North Western Med. & Surg. Jour.*, Dec., 1871.

‡ Report of a paper read before the N. Y. Society of Neurology and Electrology, *N. Y. Med. Journal*, vol. 21, p. 59.

atropia prevents or lessens the depression in the respiration and cardiac movements caused by the other two, while it contributes to their cerebral effects."

"These physiological studies are confirmed by the therapeutical results. The combination of chloral, morphia and atropia is adapted to those cases of insomnia caused by pain, or in which chloral or morphia alone merely increases the cerebral excitement—as in hypochondria, puerperal mania, etc. This combination is also indicated in cases of fatty and irritable heart. When pain is to be relieved, chloral is not so serviceable as the combination with morphia and atropia. The local administration—the insertion of the medicament at the site of pain—is more effective than the merely systemic impression. This is especially the case in tic-douloureux, sciatica and coccydinia, which are much more effectually treated by injections made in the neighborhood of nerves, the seat of pain. The combination of a local irritant and benumbing agent with a systemic anodyne is more curative than either used singly."

Dr. E. Chenery,* of Boston, looks upon the combination in the same way.

Dr. F. D. Lente† finds that combining a little codeia or McMunn's elixir with chloral enhances the effect of the latter.

Dr. A. P. Hayne, Inebriates' Home, San Francisco, has used the combination of chloral hydrate and bromide of potassium in a large number of instances (2,000 to 3,000), and finds that the bromide not only enhances the effect of the chloral, but acts as a guard or check upon its occasional ill effects.

Oré‡ found that two drops of a 10 per cent. solution carbonate of soda, when added to 15 gr. of chloral in one drachm of water, would make the solution alkaline. This solution was intended and used for intra-venous injection but it has since been used,§ although not extensively, in giving the drug by the stomach. It prevents irritating local effect, and seems to add to the rapidity and intensity of its action.

---

* *Boston Med. & Surg. Journal*, 1874, p. 634.

† Personal communication.

‡ *Bulletin Gén. de Thérap.*, April, 1875.

§ Les Testa, *Gaz. Méd. de Bordeaux*, 1875, 1876.

Dr. Saml. E. Wills,* of Earlsville, Maryland, finds that, in some cases, chloral does not act well, and believing that this is due to excessive acidity of stomach, gives 20 or 30 grains of bicarbonate of soda or potash, and sleep always follows.

Chloral dissolved in cod liver oil was, I believe, first used by Dr. J. Hughes Bennett,† who tried it in a case of phthisis. He found that the combination produced vomiting, while either oil or chloral alone did not do so.

By others‡ the chloral is said to render the oil more palatable, check cough, improve appetite and check night-sweats. The proportion is as follows :

| | | |
|---|---|---|
| ℞ | Chloral hydrat.................... | gr. 10 vel 20 |
| | Ol. Morrhuæ.................... | gr. 190 |

Dr. John Barclay§ noticed the peculiar fact that when chloral hydrate and iodide of potassium were given together, in small doses, no less than ten out of fifteen patients were affected with iodism by the first or second dose of the mixture.

"Assistant Surgeon,"¶ having seen Dr. Barclay's article, put twenty patients upon this combination, but in no instance was there any iodism produced, although the medicine was given three times a day for three days.

I have tried this experiment, varying the doses of the drugs, in a number of instances, but without any appreciable increase in the effect of the iodide.

Chloral hydrate and sweet spirits of nitre are said to be incompatible,|| the mixture containing them becoming acrid and pungent in a few hours' time. This I have also tried, but have failed to confirm.

As chloral hydrate is quite irritating to the buccal, pharyngeal and gastric mucous membrane, whether in a simple aqueous or a syrupy solution, it is best to eat a little something, say a cracker, before taking the dose.** This has been spoken of in Dr.

---

* Personal communication.

† *Edinburgh Med. Journal*, June, 1870.

‡ (*Med. Times & Gaz.*,) *N. Y. Med. Record* 1871, p. 419. Frossini, Dujardin, Lissonde, *Bulletin Gén. de Thérap.*, vol. 87, p. 189.

§ *Lancet*, Sept. 21, 1872.

¶ *Lancet*, Oct. 19, 1872.

|| *Druggists' Circular*, Nov., 1877.

** Testa, op. cit.

Squibb's letter. The reason may be seen and appreciated by anyone.

A very pleasant formula is given by F. F. Harvey,* Assistant Surgeon, U. S. A. He gives it in mint water or syr. tolut, with the addition of tinct. cardamom. This "covers the taste well, and leaves the stomach in a good condition to digest food—an important matter in delirium tremens."

Limousin,† availing himself of the fact that chloral becomes liquid at a comparatively low temperature (114.8° F.), reduced it to liquid form, and ran it into capsules, where it speedily hardened. These capsules fully protected the drug from the air, for a reasonable length of time, and the effect on the system was found to be excellent. They were satisfactorily used by Liégeois and Mauriac with good results. These capsules, when the drug had solidified, were hermetically sealed after the method of Viel, of Tours. Soft capsules did not prove satisfactory. The amount of chloral in each capsule varied from 0.30 to 0.40 grams. In concluding his remarks, Limousin states that the alcoholate of chloral is much easier to manipulate and preserve than the hydrate.

Dr. H. Fly Smith‡ found that chloral, when given in camphor water, acted much more quickly and powerfully than when given in syrup.

Dr. Julius T. Hoffmann, of Chicago, Ill., and a recent writer in the *N. Y. Medical Record* (Nov. 20, 1880) say that the taste of chloral may be effectually disguised by giving it in syrup of gooseberry and adding one drop of chloroform to every grain of chloral.

*By the Rectum.*—Chloral hydrate is peculiar in that it acts with about the same rapidity and intensity when given by the rectum as when given by the mouth. Indeed, some authorities§ claim that it acts more rapidly when given by this channel. It has been found of great advantage to give it in this way in two classes of cases:

1. Those where, owing to some spasmodic or convulsive affec-

---

* *N. Y. Med. Record*, 1870, p. 328.

† *Bull. Gén. de Thérap.*, Mar. 30, 1870.

‡ *Lancet*, June, 1871.

§ Bouchut, op. cit.

tion (tetanus, puerperal convulsions), it is impossible or very troublesome to give it by the mouth, and

2. In cases where, from inflammatory or other affections of the stomach or throat, it is not deemed advisable to give the drug by the mouth, owing to the possibility of causing a local irritant action.

It may be exhibited either in the form of suppository or enema. The former is preferable in those cases where there is irritability of the rectum and a quantity of fluid cannot be tolerated.

It is a matter oftentimes overlooked by physicians, that chloral hydrate is a ready solvent for fats, so much so that solid fat becomes liquefied by contact. For this reason, cocoa butter, the usual vehicle for suppositories, is inadmissible for making them with chloral, as in such case a soft, oily mass, altogether too fluid for anything but an ointment, results. If it is desired to use a solid extract with the chloral, the difficulty is still further increased, for the little water necessary to moisten the extract before "working" it, greatly increases the fluidity of the oleaginous mixture. A writer in the *Druggists' Circular and Gazette,** after a number of experiments as to the best excipient, found that equal parts of spermaceti and oleum theobromæ have the advantage over any other. This proportion is suitable for a suppository containing ten or twelve grains of chloral. If more is used, the amount of spermaceti must be increased.

M. Catillon,† in a communication to the Société de Thérapeutique, recommends the following formula:

| | | |
|---|---|---|
| Chloral hydrate | ............................ | 1 part. |
| White wax,<br>Cocoa butter, | } aa ........................ | 2 parts. |

for plasters, suppositories or bougies.

Whidborne‡ finds a suppository mass composed of one to two drachms of chloral hydrate, made up with hard soap and honey, very useful.

---

* Feb., 1877.

† *Gazette Hebdom.*, Jan., 1879.

‡ Quoted by A. E. McRae, *Edinburgh Med. Journal*, Nov., 1871.

Paul,* who uses suppositories of chloral hydrate only for their local action in cancer, etc., employed the following as a basis:

| | |
|---|---|
| Cocoa butter ........................... | 30 grains. |
| Spermaceti ........................... | 45 " |
| Powdered charcoal ........................ | 45 " |

*Enemata.*—It is by enema that chloral is most often given by the rectum. Given in pure water, it, after a few injections, and sometimes in the first instance, produces considerable irritation.

Dr. G. de G. Griffith† finds it of great advantage to beat up the drug with one or two raw eggs, and to this add a little warm milk. He has used chloral and bromide of potassium in this way in a number of instances, without producing any irritation, and with the best effect on the disease. Dujardin-Beaumetz‡ has used the same base for exhibiting plain chloral with the best result. Leo Testa§ also speaks highly of it.

The use of chloral hydrate by rectal injection is spoken of as the best by Ernest Labbec.¶

Those diseases in which chloral hydrate has been used with special advantage, will be fully discussed in a subsequent article.

*Subcutaneously.*—Chloral hydrate was at one time extensively used hypodermically, but has long since fallen into disuse, owing to the commonly occurring accompaniment of pain, inflammation and abscess. Bartholow|| claims that when used subcutaneously, this drug has the power of relieving pain, which it has not when given by the stomach. Labbec,** on the contrary, asserts that its action is no way different by this mode of exhibition than by any other. Having tried it in several cases, I have found that it has distinct pain-relieving power, especially in the simple neuralgiæ. Given thus with morphia, the action of both drugs is increased.

Severe inflammation and abscess are not uncommon. Picöt††

---

* *Bulletin Gén. de Thérap.*, Avril, 1875.

† *British Med. Journal*, May 8, 1875.

‡ *Bulletin Gén. de Thérap.*, June 15, 1876.

§ *Gaz. Méd. de Bordeaux*, 1875, vol. 90, p. 332.

¶ Du Chloral, *Bull. Gén. de Thérap.*, 1870, T. 2, p. 330.

|| Hypodermic Medication, Phila., 1879, p. 190.

** *Bull. Gén. de Thérap.*, 1870, T. 2, p. 330.

†† *Gaz. Hebdom. Méd. et Chirurg.*, 1869, p. 776.

found that subcutaneous injection of chloral hydrate into the ears of rabbits was always followed by intense inflammation, these organs in most instances becoming gangrenous and falling off. Laborde* found severe inflammation, abscess and gangrene to follow hypodermic injections under the skin of guinea pigs and other animals. Colin† does not seem to have had the same bad results. He cautions, however, against the use of a strong solution, and advises not to inject in the neck or in the neighborhood of joints.

Namias,‡ of Venice, has used the drug in this manner frequently and with good results in supra-orbital neuralgia. He never observed eschars or abscess where a sufficiently diluted solution was employed. Liebreich,§ while not advocating this practice, says that he has never had abscess follow when using a platino-iridium syringe. Fairland,¶ staff surgeon, Lucknow, has used it hypodermically in cholera without the production of abscess. In a case of uræmic convulsions, and one of delirium tremens, in the Cook County hospital, Chicago,|| chloral hydrate was given subcutaneously and no abscess resulted.

Tizzoni and Fogliata** found, after an elaborate series of experiments, that gangrenous abscesses often resulted from the hypodermic or intra-venous injection of chloral hydrate. Drs. A. Ady,†† of West Liberty, Iowa; A. M. Campbell,†† of Mt. Vernon, N.Y.; Wm. Judkins,†† of Cincinnati; Walter Edmunds,†† of London, Eng.; G. W. Davis,†† of Chico, California, and F. L. Forsyth,†† of Providence, R. I., have seen inflammation, abscess or sloughing follow. Bouchut‡‡ does not believe that this method of giving chloral hydrate is free from danger: that of inflammation, gangrene and abscess. Sir James Simpson§§ saw slough

---

* *Archiv. Gén. de Méd.*, 1869, T. 2, p. 758.
† *Gaz. des Hopitaux*, 1874, p. 611.
‡ *Archiv. Gén. de Méd.*, 1870, T. 1, p. 240.
§ *Berlin Klin. Wochenschrift*, No. 43, 1870.
¶ *British Med. Jour.*, Jan. 27, 1877.
|| *N. Y. Med. Record*, Aug. 14, 1880.
** *Lancet*, Nov. 11, 1876.
†† Personal communication.
‡‡ Cte. Rendu, *Ac. des Sciences*, 1869, p. 966.
§§ *Edinburgh Med. & Surg. Journal*, May, 1870.

follow this method in one case, as did Giraldes,* Ogle† and Nairne‡ in several cases.

The pain, inflammation, sloughing and abscess so often seen to follow the hypodermic use of this drug, are undoubtedly due to its acidity. This may be effectually neutralized by the addition to the solution, *at the time of giving*, of carbonate of soda as recommended by Oré. One grain of carbonate of soda will effectually neutralize the acidity of 20 gr. of chloral hydrate, and while doing away with pain and subsequent inflammation, will in no way impair the activity of the drug.

In making these injections, care should be taken that a vein is not punctured (the cellular tissue of the shoulder over the deltoid being the best site for operating, the needle being introduced at a right angle to the long axis of the limb), that the chloral is fully neutralized and the solution well diluted. The plan of giving chloral by the rectum, when it cannot be given by the mouth, is so simple and efficacious that the hypodermic plan is seldom called for—chiefly in neuralgic affections. In these cases, morphia and atropia may, with advantage, be combined with the chloral, or what is better, give an injection of choral first and then one of morphia and atropia soon after, for the alkaline solution of chloral will precipitate the morphia if all are given together.

*Intra-venous Injection.*—The intra-venous injection of chloral hydrate is, in my opinion, never justifiable. Oré, the great champion of this method, still believes in it and occasionally practices it. When first brought before the profession and so ably advocated by this gentleman, it found many supporters in France and Germany, and almost none in England and America. It was used for two purposes: 1st, and chiefly, to produce profound anæsthesia; 2d, to relieve severe spasm, as in tetanus and diseases of like nature. As we know, its use by the rectum or hypodermically is quite sufficient in the latter class, and in the former we have much safer and quite as efficacious agents as chloral.

---

* *Gaz. des Hôpitaux*, 1869, p. 523.

† *Practitioner*, 1870, p. 267.

‡ *Practitioner*, July, 1875.

Successful, at first, in a few cases, deaths* soon began to result, and in 1874 the French Academy condemned the practice as dangerous and unjustifiable.

Vulpian† found, in experimenting on dogs, that death resulted in several cases, and occasionally hæmaturia. Colin‡ found, in experimenting on animals, that a strong solution, or the rapid injection of a weaker solution, caused death by syncope. Oré,§ the great exponent of this method, says that the hæmaturia observed by Vulpian in animals has never occurred in man. In this article he refers to fourteen successful cases, and makes the following statements based thereon: "An essential condition of success consists in making the puncture without laying bare the vein, and especially without dissecting or isolating the vessel. If the subject is too fat to permit of this, and the veins are scarcely apparent, a cut should be made over the vessel, sufficient to expose its outside, and the puncture he made directly, without separating the vessel from neighboring tissues."

The strength of the solution is 1 to 4, sometimes 1 to 6. "Whatever the solution, our observations teach us that no less than 5 grams are needed to produce anæsthesia, and from 5 to 8 grams are necessary to obtain sufficient anæsthesia to permit of the performance of a capital operation. Insensibility of the cornea is taken as a test for general anæsthesia. One gram a minute may be injected until the necessary amount is introduced. The canula should not be left in the vein too long, or a clot will form."

He sums up by saying: "1. There is never any respiratory trouble. 2. Insensibility, varying according to the amount of the dose, is produced. 3. There is no preceding period of excitement. 4. There is never any vomiting. 5. The anæsthesia is always followed by deep sleep, which is calm and regular, and which lasts from ten to twenty-four hours, thus doing away completely with all the evil after-effects of the operation. 6. There is never phlebitis, clots or hæmaturia, if the injection is properly made."

---

* Lande, *Med Times & Gazette*, Jan. 16, 1876.

† *Bulletin Gén. de Thérap.*, vol. 86, 1874, p 544.

‡ *Bulletin Gén. de Thérap.*, vol. 87, 1874, p. 80.

§ *Bulletin Gén. de Thérap.*, vol. 87, 1874, p. 468.

A case of hydrophobia is reported* in which 13 grams were injected one day and 20 grams the next. Death occurred on the second day from orthopnœa. The veins were found in good condition, there being neither thrombi or emboli found.

The intra-venous use of chloral for the purpose of producing sufficient anæsthesia to permit the performance of capital and other operations, has been fully tried† and by the majority of operators condemned.

Sigs. Tizzoni and Fogliata‡ conclude, from forty experiments, "That chloral injected into the veins does not act as a true anæsthetic, but simply as a strong hypnotic. That it is very dangerous, and that the evil effects, when they show themselves, cannot be combated; also, that phlebitis is easily induced."

Dr. Noir,§ of Brioude, France, amputated a leg, the patient being fully anæsthetized by means of the intra-venous injection of 75 grains of chloral. Alarming collapse occurred upon waking, and was followed by violent delirium. He does not favor its use.

Experiments on animals, to some of which I have already referred, prove to us that the danger from syncope is quite as great as that from clot. This is further clearly demonstrated by the experiments of V. Chiorne.¶ He found that phlebitis was not an uncommon occurrence, and has seen animals die from arrest of the heart's action in diastole.

Francis, Frank and Troquart, in experimenting in Marcy's laboratory, found that the intra-venous injection of large doses of a concentrated solution of chloral caused arrest of heart and

---

* Rép. de Pharm., 1874.

† Landrin, *Archives Gén.*, 1869, T. 2, p. 755. Guerin, *Bull. Gén. de Thérap.*, vol. 86, p. 330. Bouchut, *Archiv. Gén. de Méd.*, 1869, T. 2, p. 755; *Gaz. des Hôpitaux*, 1878. p. 745. Labbée, E., *Bull. Gén. de Thérap.*, 1870, T. 2, p. 330. Deneffe and Van Wetler (*Gaz. des Hôpitaux*, 1874, p. 569), *Med. Times & Gaz.*, Oct. 1874. Bouillaud (L'Institut), *Med. Times & Gaz.*, Aug. 14, 1875. Redier (*Jour. des Sci. Méd. de Lille*), *Druggists' Circular*, Aug., 1879. Tizzoni and Togliata (*Revista Clin. di Bolog.*, Fas. 12, 1875), *Lancet*, Nov. 11. 1876. Giraldes, *Gaz. des Hôpitaux*, 1869, p. 523. Noir, *Gazette des Hôpitaux*, 1869, p. 590. Liebreich, Das Chloral Hydrat, Ein neues Hypnoticum und Anæstheticum und dessen Anweudung in der Medicin; Berlin, 1871. Valentinotti and Sylvestri, *Gaz. Obstétricale*, Feb., 1874. p. 29. Nairne, *Glasgow Med. Jour.*, July, 1875. Mauriac, *Gaz. des Hôp.*, 1870, p. 405. Oré, *Gaz. des Hôpitaux*, 1872, p. 524; 1874, p. 611. Linhart, Lande, *Bull. Gén. de Thérap.*, vol. 91, pp. 35, 82.

‡ (*Revista Clin. di Bolog.*, Fas. 12, 1875), *Practitioner*, 1877, p. 214.

§ *Gaz. des Hôpitaux*, Dec. 29, 1869.

¶ (*Lo Sperimentale*, Fasc. 1, 1876,) *Allg. Med. Cent. Zeit.*, April 29, 1876.

respiration, and show clearly that the arrest of the heart's action is due to contact of chloral with the endocardium, and not by action on cerebral centers.

Intra-venous injection of chloral hydrate, then, is dangerous from the fact that phlebitis may follow, a clot may form, and the fatal syncope may result. As has already been said, I consider the practice unsafe and unjustifiable.

*Inhalation.*—Richardson* administered chloral hydrate by inhalation. He dissolved the chloral in ether and then caused it to be inhaled. Mandl† has used cigarettes containing chloral for inhalation.

Those preparations of chloral used locally will be discussed under the head of those diseases calling for the local application of this drug.

* Quoted by Ernest Labbee, *Bull. Gén. de Thérap.*, 1870, T. 2, p. 330.

